# Baltimore Dentists in Germany

**Published:** 1892-09

**Authors:** 


### Editorial, Etc.

BALTIMORE DENTISTS IN GERMANY.—Despite the well-known fact that Germany annually sends her contingent of students to America to study dentistry at the world-renowned dental colleges of Baltimore, whose graduates are found in large numbers in the official dental directory of Germany,a campaign has for some time been carried on, particularly by the police of Berlin, against the so-called "wild American doctors" with trans-Atlantic diplomas. This has led to many curious results.

Quite a number of persons, who on their shingles announced themselves as American doctors and dentists, or "dentists approved in Baltimore," have been sentenced to fines of various amounts, some as high as 300 marks. The question of their right to such titles has been closely inquired into. Through the foreign department the police have in many cases found out that the diplomas were not acquired *rite*, that is, after a proper course of studying in an American college.

Besides the well-known Baltimore institutions to which a particular importance has been attached for long years because of the superior efficiency of its graduates practicing in the

---

*The tooth was then given the President, who removed the filling with strong ezcavators, and stated thst, in his opinion, it was more firmly held in place than would have been possible in such a cavity without the use of cement.

Fatherland, the following schools are known to be legally entitled to confer the degress, and acknowledged as such, namely, the University of Michigan, the University of Wisconsin, the Cental College of Delaware, the Collegium Dentale Philadelphiæ in Republica Pensylvaniense, the New York College of Dentistry, the Collegium Columbianum and the Collegium Medicorum Chirurgorum Novi Eboraci, (New York.)

On the other hand it is alleged that other "colleges", of which numerous diplomas are afloat, never existed. Thus persons of both sexes who, after a regular course of study at an officially authorized American institution, have received then and there the degree of doctor of dental surgery, doctor of dentistry, etc., are permitted to advertise themselves as "doctor of dental surgery, etc., of such and such university or college;" but they are not allowed to call themselves American dentist, or "doctor of dentistry approved in America," or "not approved in Germany," because the police authorities interpret this as tantamount to "assuming a medical or dental title," and the courts have in many cases taken the like view. The Baltimore colleges are really the most thorough dental colleges in the United States, and it is not surprising that the Germans should be getting more particular in this matter.—GOSSIP FROM ABROAD.—*The Baltimore Sun.*

